# Changes of uterocervical angle and cervical length in early and mid-pregnancy and their value in predicting spontaneous preterm birth

**DOI:** 10.3389/fphys.2024.1304513

**Published:** 2024-03-21

**Authors:** Miaomiao Zhang, Shuilan Li, Chao Tian, Min Li, Baofang Zhang, Hongkui Yu

**Affiliations:** Department of Sonography, Shenzhen Baoan Women’s and Children’s Hospital, Shenzhen, China

**Keywords:** cervical length, uterocervical angle, spontaneous preterm birth, transvaginal ultrasound examination, early and mid-pregnancy

## Abstract

**Objective:** To explore the feasibility of transvaginal ultrasound measurement of uterocervical angle (UCA) and cervical length (CL) in early and mid-pregnancy and evaluate their combined prediction of spontaneous preterm birth (sPTB) in singleton pregnancies.

**Patients and Methods:** This retrospective study comprised 274 pregnant women who underwent transvaginal ultrasound measurement of CL in mid-pregnancy (15–23+6 weeks); in 75 among them, CL also had been measured in early-pregnancy (<14 weeks). These 274 pregnant women were further divided into a preterm group (n = 149, <37 weeks gestation) and a control group (n = 125, >37 weeks gestation) according to delivery before or after 37 weeks, respectively. In the preterm group, 35 patients delivered before 34 weeks and the remaining 114 delivered between 34 and 37 weeks.

**Results:** The optimal threshold of CL to predict preterm birth risk in women with <37 weeks gestation was 3.38 cm, and the optimal threshold of the UCA to predict preterm birth risk in the same group of women was 96°. The optimal threshold of CL to predict preterm birth risk in women with <34 weeks gestation was 2.54 cm, while that of the UCA in the same group of patients was 106°. The area under the curve for predicting preterm birth by combining the UCA and CL measurements was greater than that by using the UCA or CL alone (*p* < 0.01). The sensitivity and specificity for predicting preterm birth at <34 weeks gestation was 71.7% and 86.4%, respectively; and the sensitivity and specificity for predicting preterm birth at <37 weeks gestation was 87.6% and 80.6%, respectively. The difference between the two groups in CL and UCA were not significant in early pregnancy (*p* > 0.01), but only in mid-pregnancy (*p* < 0.01). There was a negative correlation between UCA and gestational week at delivery (r = −0.361, *p* < 0.001) and a positive correlation between CL and gestational week at delivery (r = 0.346, *p* < 0.001) in mid-pregnancy. The proportion of deliveries at <34 weeks was highest when the UCA was >105°, and the proportion of deliveries between 35 and 37 weeks was highest when the UCA was between 95° and 105°. The proportion of deliveries at <34 weeks was highest when the CL was <2.5 cm.

**Conclusion:** The combination of UCA and CL has a better ability to predict preterm birth than either measurement alone. A more obtuse UCA or a shorter CL is associated with an earlier spontaneous preterm birth. The UCA increases from early to mid-pregnancy, while the CL decreases from early to mid-pregnancy.

## 1 Introduction

According to global statistics, more than one-tenth (15 million) of infants are born prematurely every year (Blencowe et al., 2012), and about 1 million infants die from complications of preterm birth (Perin et al., 2022). It is reported that approximately 75% of these deaths could be avoided by timely intervention measures (Iams et al., 2008). According to the gestational week of preterm birth, it can be divided into the following types: extremely preterm birth occurring before 28 weeks, accounting for about 5%, severe preterm birth occurring between 28 and 31 weeks, accounting for about 15%, moderate preterm birth occurring between 32 and 33 weeks, accounting for about 20%, and near-term birth occurring between 34 and 36 weeks, accounting for 60%–70% (Goldenberg et al., 2008). Therefore, early identification of pregnant women at risk of preterm birth is critical, which can provide them with timely follow-up and treatment (Cunningham et al., 2014).

Currently, the techniques for diagnosing risk of preterm birth include the patient’s history of preterm birth; transvaginal ultrasound; clinical signs including uterine contractions, vaginal bleeding, uterocervical angle (UCA) and cervical length (CL) (McCarty-Singleton and Sciscione, 2012). During pregnancy and delivery, the cervical tissue undergoes a series of cervical remodeling behaviors such as softening and increased compliance, which enables the cervix to better adapt to the gravity of pregnancy tissue and fetal delivery (Myers et al., 2015). Therefore, the cervix is considered an anatomical marker of the potential pathological process leading to spontaneous preterm birth (Wendy and Katherine, 2011), and transvaginal ultrasound measurement of CL or UCA has been applied in the clinical prediction of preterm birth (Dziadosz et al., 2016). However, Hiersch et al. (2016) analyzed 1,068 pregnant women at high risk of preterm birth in mid-pregnancy and found that a simple CL measurement of <3 cm could not really predict preterm birth with good specificity. Given that the measurement of UCA is closely related to factors such as gestational week, patient ethnicity, and medical history, which leads to differences in the optimal cut-off point judgment among various studies, the international range of UCA for predicting preterm birth is between 95° and 115°(Dziadosz et al., 2016; Farràs Llobet et al., 2017). There are only few studies on the optimal cut-off segment of UCA for Chinese people (Shi et al., 2018). At present, limited literature exists on the analysis of spontaneous preterm birth prediction by combining CL and UCA in a multivariate regression model in mid-pregnancy and the relationship between gestational period (early pregnancy vs. mid-pregnancy) and preterm birth. We hope that our research data can further supplement this field of study.

## 2 Materials and methods

### 2.1 Subjects

A retrospective study was conducted among 30,039 pregnant women who delivered at Shenzhen Baoan Women’s and Children’s Hospital from January 2021 to March 2023. Of these, 1,865 were born prematurely with a rate of 6.2%. Out of 1865, 149 persons who had mid-trimester CL measurements were selected as the experimental group. Age and height and weight matched 125 full term deliveries were used as controls. The study was approved by the ethical review board of our hospital.

Inclusion criteria: (1) clear and excellent cervical images; (2) complete clinical data.

Exclusion criteria: (1) multiple pregnancies; (2) congenital fetal malformations; (3) premature rupture of membranes; (4) uterine malformations.

### 2.2 Measurement of CL and UCA

**FIGURE 1:**
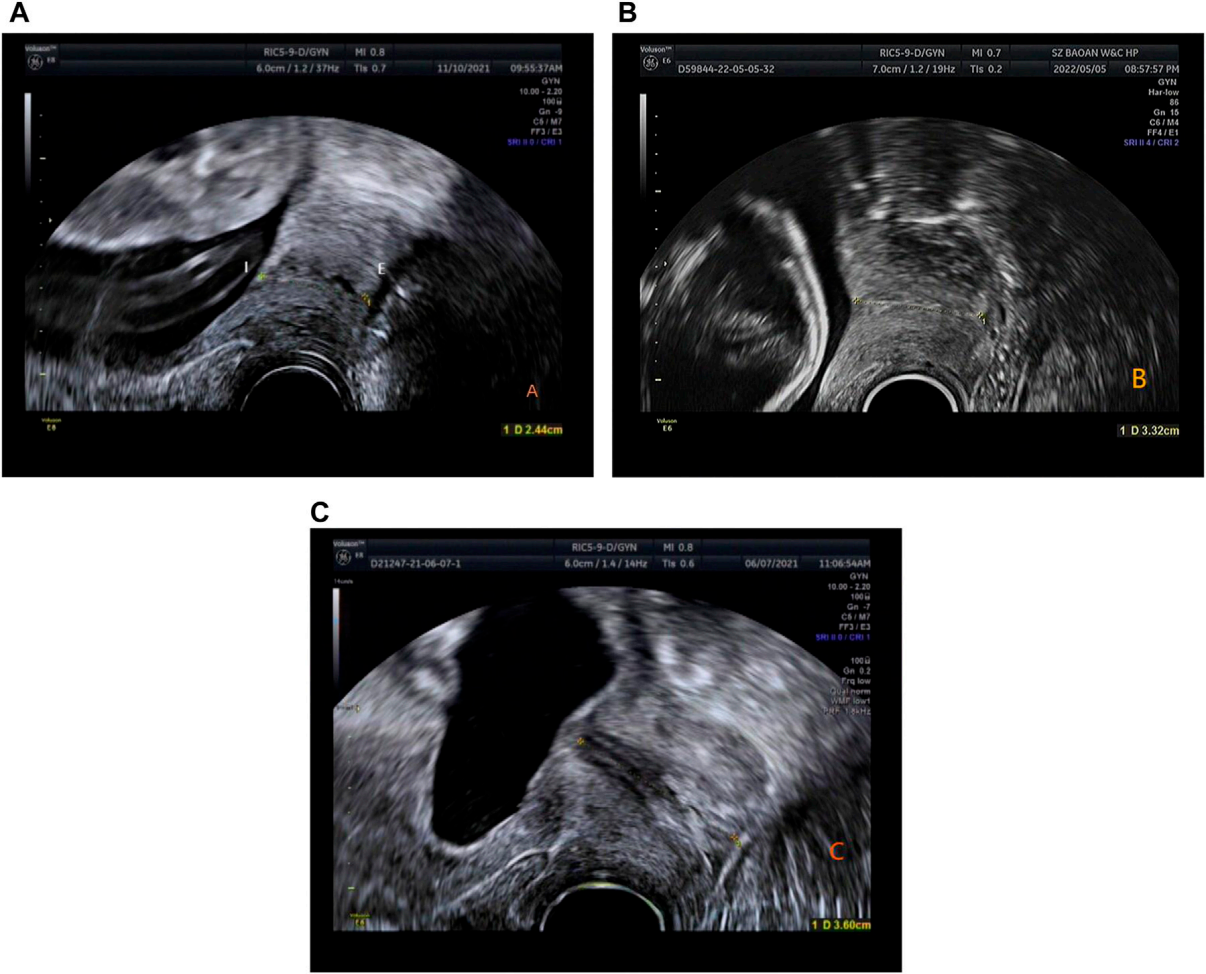
**(A)** Time of delivery <34 weeks, CL is 2.44 cm (gestation time: 23 weeks 5 days). **(B)** Time of delivery between 34 and 37 weeks, CL is 3.32 cm (gestation time: 23 weeks). **(C)** Time of delivery >37 weeks, CL is 3.6 cm (gestation time: 20 weeks 2 days).

**FIGURE 2:**
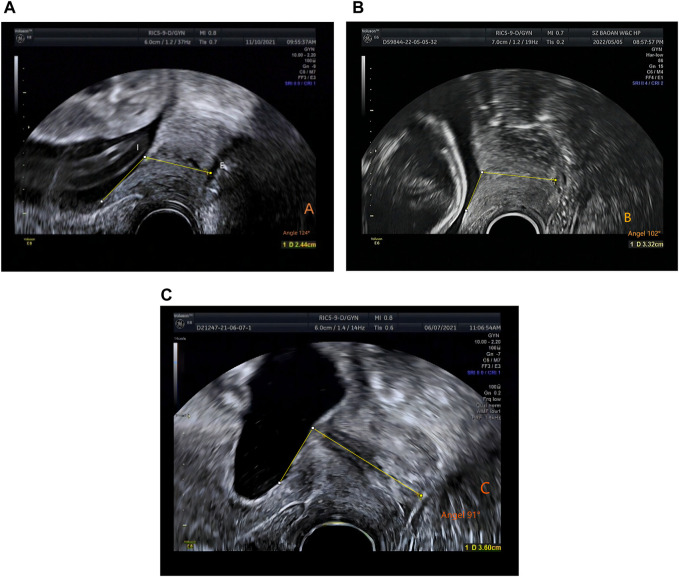
The measurement of uterocervical angle (UCA). UCA was calculated as the angle between two lines. The first line was drawn between the internal **(I)** and external ostium (os) **(E)**. The second line was drawn 3 cm parallel to the lower aspect of the anterior inner uterine wall passing through the end of the first line at the internal os. **(A)** Time of delivery <34 weeks, UCA 124° (gestation time: 23 weeks and 5 days). **(B)** Time of delivery between 34 and 37 weeks, UCA 102° (gestation time: 17 weeks). **(C)** Time of delivery >37 weeks, UCA 91° (gestation time: 17 weeks 1 day).

In this retrospective study, the sample measurement was performed by several attending ultrasound physicians with >5 years’ relevant work experience, who measured the CL. Patients were examined using a GE Voluson E8 color Doppler ultrasound diagnostic instrument with RIC5-9-D probe. The Frequency range is 4.55MHz–8.33 MHz. The patients were placed in the lithotomy position after emptying the bladder, and the CL were measured. The specific operation method was as follows: The vaginal ultrasound probe was covered with a medical disposable condom. To minimize acoustic impedance, the inner and outer layers of the condom are coated with medical sterile coupling agent. The probe was slowly moved to the vault of the vagina, and images of the lower segment of the uterus and the internal and external ora of the cervix were obtained. The cervical image occupied two-thirds of the whole image. The CL was measured by the internal and external ora of the cervix on the sagittal plane of the cervix (Kagan and Sonek, 2015) (Figure 1). ImageJ software was used by an attending ultrasound physician with more than 5 years of work experience to measure the UCA on the previously stored cervical image again, and repeated 3 times to obtain the average value. The angle between the lower segment of the uterus and the internal and external os of the cervix was considered the UCA (Sawaddisan et al., 2020) (Figure 2).

### 2.3 Statistical analysis

Data analysis was performed using R version 4.2.3 (Shortstop Beagle), count data were expressed as frequency (percentage), comparison between groups was performed by chi-square test, If the theoretical frequency was relatively small, Fisher’s exact test was used. Measurement data were tested for normality and homogeneity of variance. If variables showed normal distribution and homogeneity, they were expressed as mean ± standard deviation, and *t*-test was used for intergroup comparison. Otherwise, they were expressed as the median (interquartile range), and independent sample Wilcoxon rank sum test was used for intergroup comparison. In this study, we used linear regression to analyze the relationship between cervical angle, cervical length and weeks of labor, and logistic regression to develop a predictive model for the risk of preterm birth. The area under the receiver operating characteristic (ROC) curve (AUC) and its 95% Confidence interval (CI) were calculated. Comparisons of AUC were made using the delong test. If the AUC was >0.5 and the difference was significant compared with 0.5, the diagnostic index was considered to have some diagnostic value. The point on the ROC curve where the Jordon index (sensitivity + specificity-1) was greatest was used as the cut-off point. *p* < 0.05 was considered statistically significant, and all tests were two-sided.

## 3 Results

### 3.1 General data

There was no statistical significance in the comparison of pre-pregnancy BMI, prenatal BMI, female fetus, prior artificial abortion, hypertensive disorder, prior ectopic, anemia, thyroid disease and prior spontaneous abortion between the two groups. However, there were significant differences with respect to age, delivery week, fetal weight, *in vitro* fertilizer, diabetes, cesarean delivery repeat (*p* < 0.05) (Table 1)

**TABLE 1:** Demographic data.

Demographic data	Overall	Birth at term (>37wk)	Birth at term (<37wk)	Z/t/χ2	*p*-value^b^
	N = 274^a^	N = 125 (45%)^a^	N = 149 (55%)^a^		
Age,yr	31.63 ± 3.94	32.29 ± 3.66	31.09 ± 4.09	2.429	0.015
Pre-pregnancy BMI	22.49 (19.52, 25.61)	23.04 (19.99, 25.92)	22.15 (19.28, 25.05)	1.540	0.124
Prenatal BMI	24.16 (21.29, 27.01)	24.60 (21.60, 27.51)	23.57 (21.19, 26.85)	1.473	0.141
Delivery week	36.71 (35.57, 39.00)	39.14 (38.43, 39.86)	35.71 (33.86, 36.43)	14.156	<0.001
fetal weight	2,840.00 (2,400.00, 3,250.00)	3,260.00 (3,000.00, 3,500.00)	2,430.00 (2,115.00, 2,700.00)	12.411	<0.001
Female fetas	106 (39.4%)	50 (40.7%)	56 (38.4%)	0.147	0.701
*in vitro* fertilizer	24 (8.7%)	18 (14.4%)	6 (4.0%)	9.258	0.002
Prior artificial abortion	68 (24.7%)	37 (29.6%)	31 (20.7%)	2.923	0.087
Diabetes	73 (26.5%)	25 (20.0%)	48 (32.0%)	5.035	0.025
Prior spontaneous preterm birth	33 (12.0%)	14 (11.2%)	19 (12.7%)	0.139	0.709
Hypertensive disorder	17 (6.2%)	6 (4.8%)	11 (7.3%)	0.754	0.385
Cesarean delivery repeat	32 (11.6%)	21 (16.8%)	11 (7.3%)	5.943	0.015
prior ectopic pregnancy	7 (2.5%)	5 (4.0%)	2 (1.3%)	1.027	0.311
anemia	43 (15.6%)	23 (18.4%)	20 (13.3%)	1.327	0.249
Thyroid Diseases	25 (9.1%)	15 (12.0%)	10 (6.7%)	2.347	0.126
Prior spontaneous abortion	39 (14.2%)	16 (12.8%)	23 (15.3%)	0.360	0.549

^a^ Mean ± SD; median (IQR); n (%).

^b^ Wilcoxon–Mann-Whitney test; Two Sample *t*-test; Pearson’s chi-squared test; Pearson’s chi-squared test with Yates’ continuity correction; Fisher’s Exact Test for Count Data.

### 3.2 Changes of UCA and CL in early and mid-pregnancy

From early to mid-pregnancy, the UCA increased and the CL shortened. There was no significant statistical difference in the CL and UCA between the two groups in early pregnancy. Spearman’ correlation coefficient between the UCA and CL in early pregnancy was 0.06 (*p* = 0.608), and there was no correlation between the changes of the two variables. However, the CL was shorter and the UCA was larger in the preterm group in mid-pregnancy. Spearman’s correlation coefficient between the UCA and CL was −0.517 (*p* < 0.001) (Table 2).

**TABLE 2:** Test characteristics of the UCA and CL for the prediction of sPTB.

Parameters	Overall	Birth at term (>37wk)	Birth at term (<37wk)	Z/t/χ2	*p*-value^2^
	N = 274^1^	N = 125 (45%)^1^	N = 149 (55%)^1^		
Early-pregnancy CL	3.50 ± 0.37	3.57 ± 0.40	3.37 ± 0.26	2.267	0.026
	(n = 75)	(n = 49)	(n = 26)		
Early-pregnancy UCA	75.67 ± 15.05 (n = 75)	76.43 ± 15.63	74.16 ± 14.01 (n = 26)	0.615	0.541
		(n = 49)			
mid-pregnancy CL	3.19 (2.82,3.60)	3.61 (3.30,3.96)	2.90 (2.67,3.15)	10.996	<0.001
	(n = 274)	(n = 125)	(n = 149)		
mid-pregnancy UCA	96.73 (85.40,105.67)	85.34 (75.28, 93.30)	102.51 (97.10,111.36)	−10.793	<0.001
	(n = 274)	(n = 125)	(n = 149)		

### 3.3 The relationship between UCA and CL in mid-pregnancy and gestational week at delivery

**FIGURE 3:**
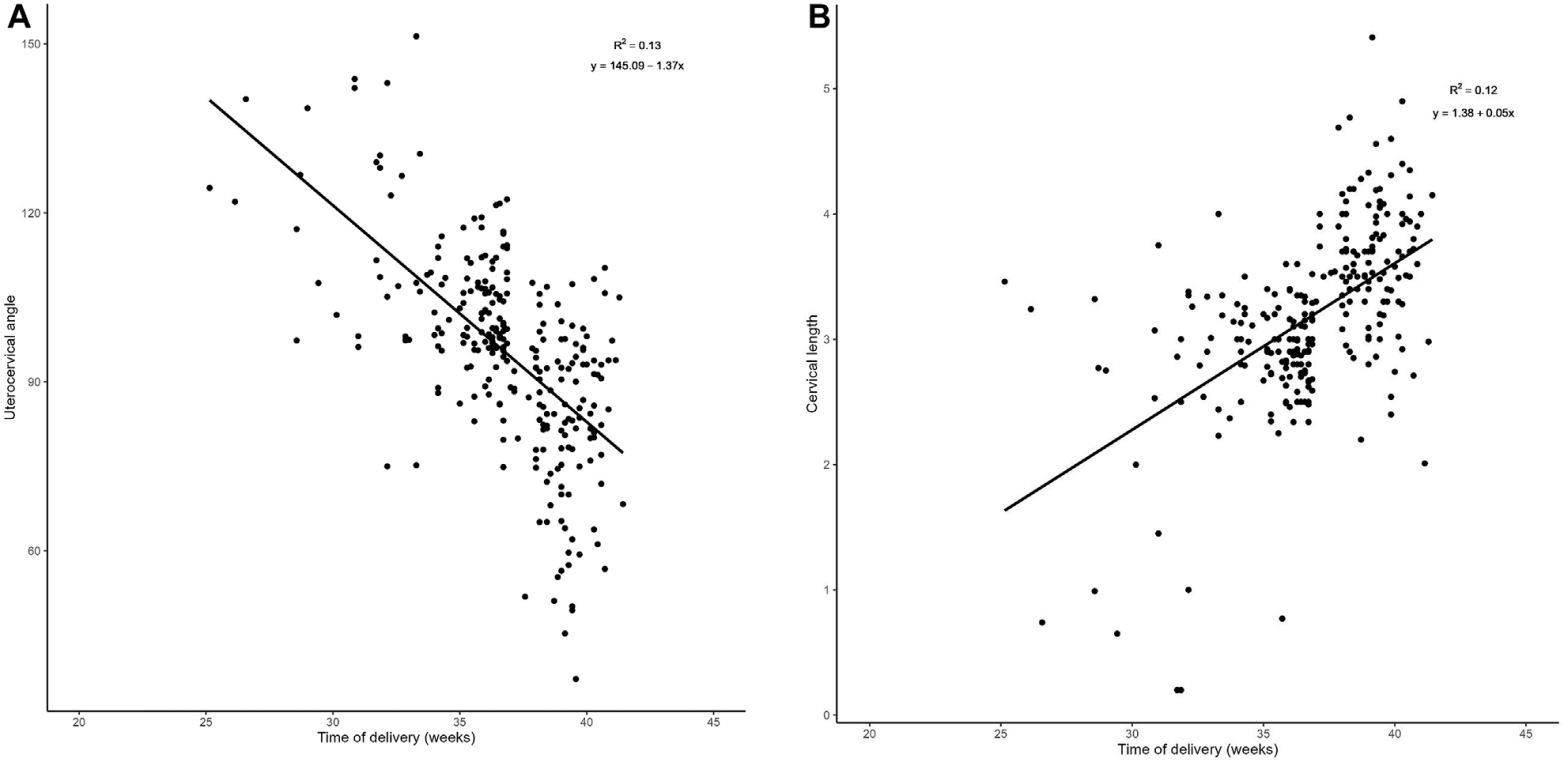
Regression analysis: **(A)**. Plot of UCA in relation to the week of labor in the middle trimester There was a negative correlation between the UCA in mid-pregnancy and gestational week at delivery (r = −0.361, *p* < 0.001).

There was a negative correlation between UCA in mid-pregnancy and gestational week at delivery (r = −0.361, *p* < 0.001) (Figure 3A), and a positive correlation between CL in mid-pregnancy and gestational week at delivery (r = 0.346, *p* < 0.001) (Figure 3B)^.^

### 3.4 Distribution of UCA and CL in different delivery gestational weeks

In Tables 3, 4, it can be seen that the proportion of deliveries that happened <34 weeks was highest when the UCA was greater than 105°, and the proportion of deliveries between 35 and 37 weeks was highest when the UCA was between 95° and 105°. The proportion of deliveries <34 weeks was highest when the CL was <2.5 cm.

**TABLE 3:** Distribution of sPTB (at < 37 weeks) and term births in women according to uterocervical angle (UCA).

UCA	Delivery at <	Delivery at 34–36+6 weeks	Delivery at ≥37 weeks
	34weeks	N = 114	N = 125
	N = 35		
≥105	26 (74.29%)	42 (36.84%)	6 (4.80%)
95–105	7 (20.00%)	54 (47.37%)	19 (15.20%)
<95	2 (5.71%)	18 (15.79%)	100 (80.00%)

Kendall’s correlation coefficient is −0.634, *p* < 0.001.

**TABLE 4:** Distribution of sPTB (at < 37 weeks) and term births in women according to cervical length (CL).

CL (cm)	Delivery at <34 weeks	Delivery at 34–37 weeks	Delivery at ≥37 weeks
≤2.5 (CM)	13 (37. 14%)	16 (13.56%)	3 (2.42%)
>2.5 (CM)	22 (62.86%)	102 (86.44%)	121 (97.58%)

### 3.5 Analysis of the efficacy graph of CL and UCA for predicting spontaneous preterm birth

**FIGURE 4:**
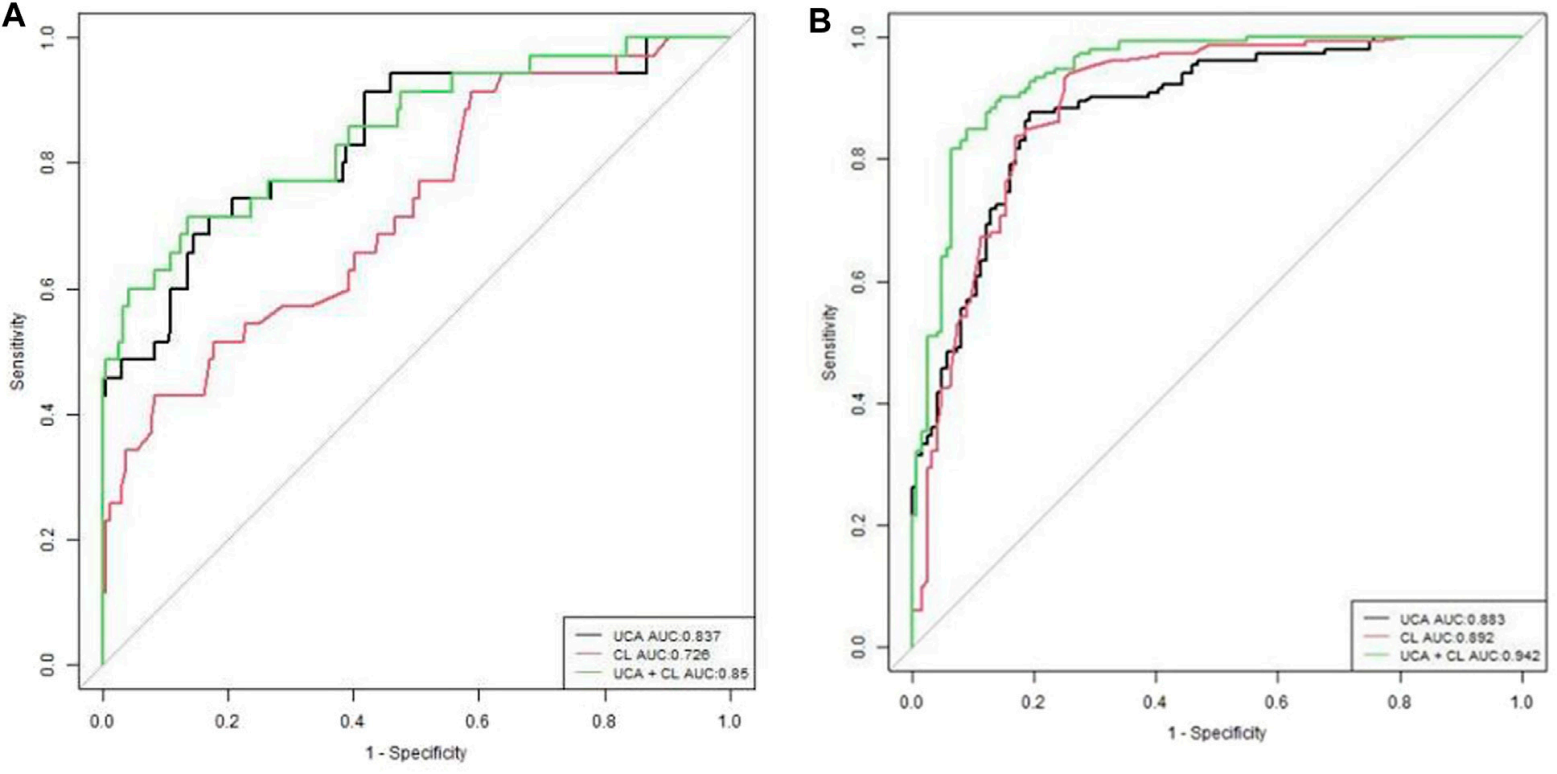
Receiver operating characteristic curves of evaluating UCA, CL, and gestational age at delivery **(A)**. UCA and CL for sPTB <34 weeks, **(B)**. UCA and CL for sPTB <37 weeks.

In Figure 4A of the efficacy graph of CL and UCA for predicting spontaneous preterm birth, it can be seen that the area under the ROC curve (AUC) for predicting preterm birth (<34 weeks) by CL was 0.726 (0.633–0.819). The threshold value was 2.54 cm, corresponding to a sensitivity, specificity, positive predictive value, and negative predictive value of 42.9%, 91.7%, 42.9%,91.7%, respectively. The AUC for predicting preterm birth (<34 weeks) by UCA was 0.837 (95% confidence interval [CI]: 0.757–0.917), and the threshold value was 106°, corresponding to a sensitivity, specificity, positive predictive value, and negative predictive value of 71.4%, 83.1%, 37.9%, and 95.3%, respectively. The AUC for predicting preterm birth (<34 weeks) by combining the CL and UCA was 0.85 (0.773–0.927), and the sensitivity, specificity, positive predictive value, and negative predictive value were 71.4%, 86.4%, 43.1%, and 95.4%, respectively.

In Figure 4B of the efficacy graph of CL and UCA for predicting spontaneous preterm birth, it could be observed that the optimal threshold for predicting preterm birth risk (<37 weeks) by CL was 3.38 cm, with an AUC of 0.892 (0.851–0.932), and the sensitivity, specificity, positive predictive value, and negative predictive value were 93.5%, 75%, 82.2%, and 90.3%, respectively. The optimal threshold for predicting preterm birth risk (<37 weeks) by UCA was 96°, with an AUC of 0.883 (0.843–0.922). The sensitivity, specificity, positive predictive value, and negative predictive value were 87.6%, 80.6%, 84.8% and, 80.4%, respectively. The AUC for predicting preterm birth risk (<37 weeks) by combining the UCA and CL was 0.942 (0.914–0.969), and the sensitivity, specificity, positive predictive value, and negative predictive value were 85%, 91.1%, 92.2%, and 83.1%,respectively.

#### 3.5.1 Statistical analysis of AUC

For preterm birth occurring <34 weeks of gestation, the combination of UCA and CL predicted preterm birth better than CL alone, with a statistically significant difference (*p* < 0.01). The UCA predicted preterm birth better than CL, with a statistically significant difference (*p* < 0.01). The efficacy of predicting preterm birth by combining UCA and CL was the same as that by UCA alone, with no statistical significance (*p* > 0.01) (Figure 4A; Table 5).

**TABLE 5:** Statistical analysis of area under the curve (AUC) in Figure 3A, B.

Delivery at <34 weeks	*p*-value	Delivery at <37 weeks	*p*-value
UCA (0.837)vs. CL (0.726)	0.046	UCA (0.883) vs. CL (0.892)	0.746
UCA(0.837)vs. UCA + CL (0.85)	0.395	UCA(0.883) vs.UCA + CL (0.942)	<0.001
CL (0.726) vs. UCA + CL	0.005	CL vs. UCA + CL (0.942)	<0.001

The equation using the joint diagnostic model is: Log (p/1−p) = −9.189 + 0.095*UCA−0.904 ×CL.

For preterm birth occurring <34 weeks of gestation, a combination of UCA and CL predicted preterm birth better than either of these two indicators alone, with a statistically significant difference (*p* < 0.01). The efficacy of predicting preterm birth by UCA and CL was the same, with no statistical significance (*p* > 0.01) (Figure 4B; Table 5).

## 4 Discussion

This study showed that the combination of UCA and CL had the best effect in predicting preterm birth according to the efficacy graph. As pregnancy progressed from early to mid-stages, the UCA increased significantly and the CL shortened significantly in the preterm group. A more obtuse UCA or shorter CL was associated with a more premature spontaneous preterm birth.

Previous studies by Dziadosz et al. (Dziadosz et al., 2016) showed that the optimal threshold for CL was 2.5 cm with a specificity and negative predictive value of 98% and 96%, respectively, when preterm birth occurred<34 weeks. Comparing only single indicators, they also shows UCA was a better predictor of preterm labor than CL in predicting spontaneous preterm birth <34 weeks. Our conclusion was consistent with theirs. We found that the efficacy of UCA was greater than that of CL in predicting preterm birth <34 weeks, which was consistent with Rasha E. Khamees (Khamees et al., 2022) and Dziadosz et al.‘s (Dziadosz et al., 2016) studies. This suggests that our results are reproducible. Our optimal threshold for predicting preterm birth by UCA was consistent with the studies of Dziadosz et al. (2016) and Kumar et al. (Kumar et al., 2022), but our specificity was the highest. The specificity was 83.1% and the specificity was 80.6%, which indicates that our research results had the greatest ability to screen out patients who were not preterm, and strengthened the previous research results of Dziadosz et al. (Dziadosz et al., 2016) and Kumar et al. (Kumar et al., 2022).

The optimal threshold for CL was 3.38 cm, which was the same as that obtained by Luechathananonr et al. (2021), When preterm birth occurred<37 weeks. This is likely because our population was a predominantly Asian population, and the literature suggests that the CL of pregnant Asian women was different from that of Western women, which may be related to body mass index (BMI), height, and/or ethnicity. However, comparing single indicator alone, Luechathananonr et al. (2021) reported CL had more advantages than UCA in predicting spontaneous preterm birth<37 weeks.Our research shows UCA has no advantage over CLfor predicting spontaneous preterm labor <37 weeks. Either one of them is a good predictor of preterm labor <37 weeks.This result conflicts with Luechathananonr et al., which we will investigate further later.

In addition to drawing the above conclusions, our study also showed that the combination of UCA and CL had application and promotion value in screening our pregnant women who may have preterm birth.

Dziadosz et al. (Dziadosz et al., 2016) also concluded that the efficacy of predicting preterm birth by combining UCA and CL (including <37 weeks and <34 weeks) was higher than that by single indicators. However, their study did not provide the specific AUC value, rather they only calculated the specificity and negative predictive value. In our study, we calculated not only the sensitivity, specificity, positive predictive value, and negative predictive value but also the specific AUC value of the ROC curve. Hence, we believe our results are more complete and clinically meaningful.

We analyzed that as the pregnancy progressed from early to mid-stages, the UCA increased significantly and the CL shortened significantly in the preterm group. Spearman’s correlation coefficient between the UCA and CL in mid-pregnancy was −0.517 (*p* < 0.001), which was similar to Dziadosz et al.‘s study (Dziadosz et al., 2016) (correlation coefficient: −0.05 [*p* < 0.05]) and Kumar et al.‘s study (correlation coefficient: −0.264 [*p* < 0.001]), (Kumar et al., 2022),suggesting that the UCA increased significantly while the CL decreased significantly. According to Sochacki-Wojcicka et al. (2015) and House et al. (House et al., 2013), the cervix bears pressure from the surrounding pelvic organs and increased uterine gravity and load during pregnancy. An acute angle of the cervix can buffer these loads transmitted to the cervix, maintaining normal morphology of the cervix. An obtuse UCA may transmit these loads to the cervix, causing dilation of the cervical canal, leading to preterm birth. At the same time, Dziadosz et al. (Dziadosz et al., 2016) also pointed out that the same force acting on a blunt cervical canal may more likely cause the cervical canal to open. Therefore, the UCA changes from an acute angle in early pregnancy to an obtuse angle in mid-pregnancy, leading to preterm birth. However, the UCA of pregnant women who delivered at term remained acute from early to mid-pregnancy. This is because the acute angle forms a “inverted triangle support” in the lower segment of the uterus to support the pressure from the top, thus reducing the pressure and maintaining its normal shape (Yao et al., 2014; Yoshida et al., 2014). In this study, the change of CL length in early pregnancy was consistent with that of Thain et al. (2020), who found that there was no statistical significance in the change of CL in early pregnancy in the Asian population. However, in mid-pregnancy (18–22 weeks), the CL of the preterm group was lower than that of the control group, which was also similar to the study by Shi et al. (Shi et al., 2018). The CL of the preterm group was relatively shorter than that of the term group (3.09 cm vs.3.78 cm). There was no significant statistical difference in the UCA in early pregnancy. We speculate that the underlying mechanism is that in early pregnancy, the weight of the uterus is not large, and the cervix bears relatively less load, so the cervical function remains normal. With fetal growth, the load on the uterus increases, and at the same time, the smooth muscle or elastic fiber of the cervix reduces that, in turn, reduces cervical compliance, and resists deformation caused by collagen fiber disorder and a 5% increase in the water content. The cervix of preterm patients softens prematurely, leading to cervical shortening (Myers et al., 2009; House et al., 2013).

Our study reviewed not only the previous studies on UCA and CL in mid-pregnancy but also studies on UCA and CL in early pregnancy and compared the changes in these two stages. By studying these two stages of UCA and CL, we found that when pregnant women’s UCA changed from acute angle in early pregnancy to obtuse angle in mid-pregnancy, we should be alert to preterm birth. We observed that there was no significant statistical difference in CL and UCA between the two groups in early pregnancy. The UCA of pregnant women at this stage was acute and the CL was greater than 3.37 cm. Spearman’s correlation coefficient between the UCA and CL in early pregnancy was 0.06, *p* = 0.608, indicating that there was no correlation between the changes of these two variables. T here was no regularity in early pregnancy, and cervical softening started from mid-pregnancy (Iams et al., 1996). Therefore, we speculate that there is no difference in the softness and toughness of the cervix among all pregnant women at this stage, and there is no deformation and remodeling. This conclusion is partly consistent with Conoscenti et al. (2003), whose study showed that measuring CL in early pregnancy had no significance for predicting preterm birth. We think that measuring CL alone in early pregnancy has no significance, rather it needs to be analyzed together with CL in mid-pregnancy.

This study has some limitations. First, this was a retrospective study, and the subjects were singleton pregnant women with gestational age <25 weeks, which may have led to selection bias. Second, there were very few samples of patients who had both CL and UCA measurements in both early and mid-pregnancy. In the future, we plan to conduct prospective studies and increase the sample size of patients who have both cervical examinations in both stages of pregnancy. Finally, this study was a single-center study, and the optimal cut-off value selected may not be representative enough.

In summary, measuring the UCA combined with CL is more accurate than measuring the CL or UCA alone for predicting preterm birth risk, which is worthy of further investigation and clinical application. If transvaginal ultrasound shows that CL is short especially less than 2.48 cm or the UCA is obtuse, especially larger than 106° in mid-pregnancy, clinicians should be alerted to a high risk of preterm birth less than 34 weeks.

## 5 Scope statement

We believe that our study is a significant contribution because we identified the combination of uterocervical angle (UCA) and cervical length (CL) has a better ability to predict preterm birth than cervical length or uterocervical angle alone. A more obtuse uterocervical angle or a shorter cervical length is associated with an earlier spontaneous preterm birth. The uterocervical angle increases from early to mid-pregnancy, while the cervical length decreases from early to mid-pregnancy. Further, we believe that this paper will be of interest to the readership of your journal because it matches your researches topics about aging in female infertility and pathologic pregnancy.

## Data Availability

The original contributions presented in the study are included in the article/Supplementary material, further inquiries can be directed to the corresponding author.
